# Evaluation of the Selenotranscriptome Expression in Two Hepatocellular Carcinoma Cell Lines

**DOI:** 10.1155/2015/419561

**Published:** 2015-06-23

**Authors:** Stefano Guariniello, Giovanni Di Bernardo, Giovanni Colonna, Marcella Cammarota, Giuseppe Castello, Susan Costantini

**Affiliations:** ^1^Dipartimento di Biochimica, Biofisica e Patologia Generale, Seconda Università degli Studi di Napoli, 80138 Napoli, Italy; ^2^Dipartimento di Medicina Sperimentale, Seconda Università degli Studi di Napoli, 80138 Napoli, Italy; ^3^Servizio di Informatica Medica, Azienda Ospedaliera Universitaria, Seconda Università di Napoli, 80138 Napoli, Italy; ^4^CROM, Istituto Nazionale Tumori “Fondazione G. Pascale”, IRCCS, 80131 Napoli, Italy

## Abstract

Hepatocellular carcinoma (HCC) is the most common type of liver cancer and is still one of the most fatal cancers. Hence, it needs to identify always new putative markers to improve its diagnosis and prognosis. Since the selenium is able to fight the oxidative damage which is one of the major origins of cell damage as well as cancer, we have recently focused our attention on selenoprotein family and their involvement in HCC. In the present paper we have carried out a global analysis of the selenotranscriptome expression in HepG2 and Huh7 cells compared to the normal human hepatocytes by reverse transcription-qPCR (RT-qPCR). Our data showed that in both cells there are three downregulated (DIO1, DIO2, and SELO) and ten upregulated (GPX4, GPX7, SELK, SELM, SELN, SELT, SELV, SEP15, SEPW1, and TrxR1) genes. Additionally, interactomic studies were carried out to evaluate the ability of these down- and upregulated genes to interact between them as well as to identify putative HUB nodes representing the centers of correlation able to exercise a direct control over the coordinated genes.

## 1. Introduction

Hepatocellular carcinoma (HCC) is a common cancer with more than half a million new cases annually worldwide. Its incidence is increasing dramatically and it is due to many different risk factors such as hepatitis B (HBV) or C virus (HCV) infection, alcohol-induced liver disease (ALD), nonalcoholic fatty liver disease (NAFLD), primary biliary cirrhosis, exposure to environmental carcinogens (particularly aflatoxin), or even type 2 diabetes and obesity [1–4].

Even if there were some advances in HCC diagnosis and management, this cancer is still fatal because the patients survive less than 8 months, and the only curative modalities are liver transplantation, surgical resection, or local ablation [5, 6]. Therefore, it is necessary to identify always new putative markers to improve the HCC prognosis.

Recently we have applied the microarray technology to compare gene expression profiles associated with HCC, in HepG2 cellular line (as HCC model without viral complications and gene mutations) and human hepatocyte cells [7] by confirming few differentially expressed genes between HCC and normal hepatocytes cells by reverse transcription-qPCR analysis.

Since some studies evidenced the role of selenium (Se) for assisting cells to resist oxidative damage that is a major cause of cellular damage and is implicated as a key factor in the early stage of cancer [8].* In vivo*, Se is primarily present as selenoproteins to maintain the balance of the cellular redox state. In particular, 25 selenoproteins have been found in humans [9]. Most of them play important roles in detoxification, redox regulation, viral suppression, and immune-system protection [10], even if the biological functions of some newly identified selenoproteins still remain unknown.

We have recently been focused on some selenoproteins and their involvement in HCC and evaluated the expression of selenium binding protein-1 (SELENBP1), which incorporates exogenously Se [11, 12]. In detail, we evidenced the downregulation of SELENBP1 in the liver tissue of HCC patients and the association of its gradual loss with an increased malignant grade [11, 12]. Recently, we evidenced for the first time also the upexpression of SELM in HCC liver tissues by immunohistochemistry [13].

In the present paper we have carried out the analysis of the global expression of the selenotranscriptome family in two hepatocellular carcinoma cell lines (HepG2 and Huh7) compared to the normal human hepatocytes by means of the RT-qPCR analysis to identify new marker(s) for HCC prognosis (or diagnosis). Then, the interactomic studies were performed on these genes to evaluate their ability to interact between them and to identify the HUB nodes playing the important role in direct control over the coordinated genes.

## 2. Methods

### 2.1. RNA Preparation and Reverse Transcription-qPCR (RT-qPCR) Analysis

Total RNA from hNHEPS human hepatocytes (Lonza, Basel, Switzerland), HepG2, and Huh7 (Lonza, Basel, Switzerland) was obtained using the TRizol Reagent (Invitrogen, Milan, Italy) following the manufacturer's instructions. Each total RNA sample was treated with the DNA-free kit according to the manufacturer's instructions (Ambion). RNA samples were quantified using a NanoDrop ND-1000 spectrophotometer (Thermo Scientific, Wilmington, DE). The mRNA levels of the analysed genes were measured by a RT-qPCR amplification procedure that was previously reported [7, 14]. The primer sequences of 25 selenoprotein mRNAs are provided in Table 1. Relative quantities were calculated by the ΔΔCq method using the 18S rRNA as housekeeping gene for normalization. Statistical analyses (paired Student's *t*) were performed using Prism software (Graphpad Software, La Jolla, CA, USA). Significant differences in relative gene expression between hepatocytes and HepG2 or Huh7 are marked by (^*∗*^ *p*-value < 0.05), (^*∗∗*^ *p* value < 0.01).

### 2.2. Bioinformatics Analysis

Network analysis was performed by Ingenuity Pathway Analysis (IPA) program and using the same procedure reported in our recent paper [7]. In detail, IPA builds and explores transcriptional networks to identify regulatory events that lead from signaling events to transcriptional effects.

## 3. Results and Discussion

### 3.1. RT-qPCR Evaluations on HepG2, Huh7 and Normal Hepatocyte Cells

**Figure 1:**
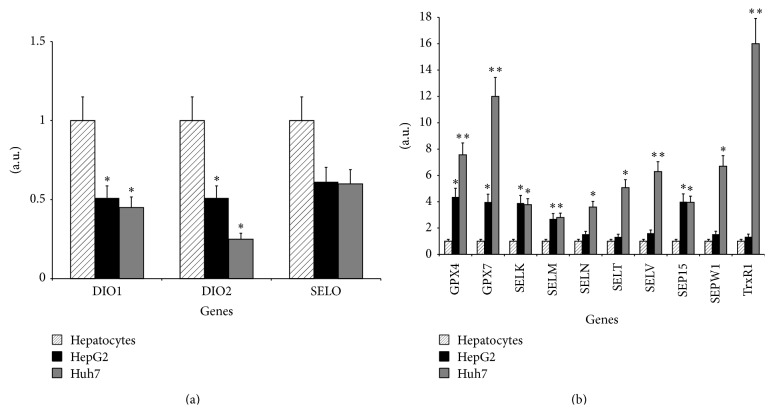
Expression of selenoprotein genes analyzed by means of RT-qPCR. The average value for the expression of these genes was obtained from three independent experiments. (a) shows the downregulated genes in HepG2 and Huh7 versus normal hepatocytes (DIO1, DIO2, and SELO) while (b) shows the ten upregulated genes (GPX4, GPX7, SELK, SELM, SELN, SELT, SELV, SEP15, SEPW1, and TrxR1). In each reaction, the expression levels were normalized to the average of the control gene (18S rRNA) and expressed as arbitrary units. The mRNA levels in hepatocytes, HepG2, and Huh7 were evaluated by using the ΔΔCt method. Significant differences in relative gene expression between hepatocytes and HepG2 or Huh7 are marked by (^*∗*^ *p* value < 0.05) and (^*∗∗*^ *p* value < 0.01).

The gene expression profiles of HepG2, Huh7, and normal hepatocyte cells by means of RT-qPCR have shown that in the two HCC cell lines there were three downregulated genes (DIO1, DIO2, and SELO) (Figure 1(a)) and ten upregulated genes (GPX4, GPX7, SELK, SELM, SELN, SELT, SELV, SEP15, SEPW1, and TrxR1) (Figure 1(b)). In detail, two of the three downregulated genes showed a statistically significant difference between HepG2 and Huh7 versus hepatocytes. On the other hand, in the group of the ten upregulated genes five of them (GPX4, GPX7, SELK, SELM, and SEP15) have shown a statistically significant upregulation in HepG2. All the other twelve selenotranscripts have appeared unchanged (*data not shown*).

It is important to underline that differences in the gene expression found for HepG2 and Huh7 could be due to differences between these two liver cancer cell lines. Both cell lines were epithelial in origin, from patients with no history of HCV and HBV infection [15]. In particular, HepG2 cells originated from liver tissue of a 15-year-old Caucasian American male affected by hepatoblastoma whereas Huh7 cells originated from a liver tumor of a 57-year-old Japanese male. However, Huh7 cells are well differentiated and recent studies have also shown that the Huh7 cell line is associated with low expression of cytokeratin 8/18 (CK8/18), while HepG2 cell line has expression of CK8/18 similar to that of normal hepatocytes [16]. In addition, HepG2 cells carry wild-type p53, whereas Huh7 cells show a high level of p53 with a constitutive mutation A:T → G:C at codon 220 and are characterized by a more malignant phenotype [16]. In detail, it is important to underline that the p53 gene is a tumor suppressor which plays an important role in the control of the normal cell cycle and, thus, is a key factor in apoptosis induction in response to chemotherapy [17]. Therefore, mutations in this protein normally result in the inability of p53 to effectively interact and to bind DNA, as well as the inactivation of residual normal forms of the protein expressed in the cells, thus preventing transcriptional activation of genes involved in cell cycle arrest and apoptosis [17]. Consequently, this means that Huh7 cells are more aggressive than HepG2 and present a more enhanced inflammatory status.

However, it has been published that SEPW1 is strictly correlated with p53; in fact, it is implicated in oxidative modifications of p53, and its knockdown induces cell cycle arrest by increasing p53 [18]. Moreover, SEPW1 presents a thioredoxin-like domain as well as SELN, SELT, SELV, and TrxR1 and all these five selenoproteins play an important role in the regulation of the redox signal [18]. Therefore, this can explain why the Huh7 cells showed a higher level of these five genes compared to HepG2 cells.

### 3.2. Network Analysis

**Figure 2:**
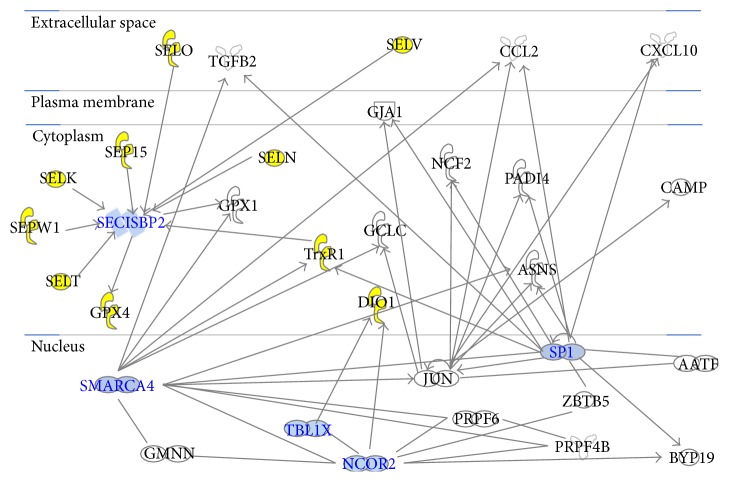
Network analysis: down- and upregulated genes are evidenced by yellow symbols, HUB nodes by cyan symbols, whereas all other genes by white symbols.

We have used the IPA algorithm to study the correlation between down- and upregulated genes.  Figure 2 shows that three downregulated (DIO1, DIO2, and SELO) and eight upregulated (GPX4, SELK, SELT, SELV, SEP15, SELN, SEPW1, and TrxR1) genes are connected in the same network named “amino acid metabolism, protein synthesis, and small molecule biochemistry” that presents some nodes (HUB nodes) that bind to our selenoprotein mRNAs: SMARCA4 (SWI/SNF related, matrix associated, actin dependent regulator of chromatin, subfamily a, member 4), SP1 (specificity protein 1), SECISBP2 (sec insertion sequence binding protein), NCOR2 (nuclear receptor corepressor 2), and TBL1X (transducin beta-like protein 1X).

Analyzing the correlations between down- and upregulated genes by means of the IPA algorithm (Figure 2) we have found that SECISBP2 (SECIS binding protein 2) binds to SELO, SELK, SELV, SEP15, GPX4, SELN, SELT, SEPW1, and TrxR1. It is a protein-coding gene that controls the incorporation of selenocysteine into proteins [19]. Indeed, TrxR1 interacts with SMARCA4 [20] and SP1 [21]. SMARCA4 is a gene encoding components of the chromatin remodeling complex by constituting the third most frequently altered class, as identified in a recent exome screening [22]. In the literature, it is reported that a high percentage of HCC tumors has a mutation in genes related to the chromatin remodeling, and, hence, this suggests a possible contribution of this pathway to hepatocyte tumorigenesis [22]; in fact, this gene was found upregulated in HCC cells [23]. Thus, the pivotal role of SMARCA4 in the selenonetwork as well as its propensity to mutate suggest that the progression of HCC might be much more complex and elusive than believed, and, hence, future researches need to unravel this important point [24].

On the other hand, SP1 is a zinc finger transcription factor that binds to GC-rich motifs of many promoters. It is involved in many cellular processes, including cell differentiation, cell growth, apoptosis, immune responses, response to DNA damage, and chromatin remodeling. SP1 plays a role in the recruitment of SMARCA4 on the c-FOS promoter and acts in synergy with other transcription factors such as NF-kB and RELA subunit by binding its specific binding sites [25]. Since both the NF-kB and the RELA play a pivotal function in the regulation of the inflammation, we reasoned that inflammation bridges immunity, HCC, and seleno-mRNAs. Moreover, in the literature it is reported that SP1 upexpression leads to p53-dependent apoptosis in cancer cells evidencing the correlation between SP1 and p53. This finding evidences the linking between the seleno-mRNAs, the HUB genes, and p53, and, hence, this findings can explain because we found a different expression of some seleno-mRNAs between HepG2 and Huh7 cells that have wild-type and mutated p53, respectively [26].

In addition, as it can be seen in Figure 2, SMARCA4 correlates with SECISBP2 through GPX1, thus suggesting a stringent correlation between SMARCA4, SP1, SECISBP2, and many other mRNAs. Therefore, this can explain their upregulation and confirm their possible involvement in development and/or progression of HCC.

Figure 2 shows also that DIO1 interacts with NCOR2 and TBL1X. In particular, NCOR2 is a transcriptional coregulatory protein with several nuclear receptor-interacting domains, devoted to assist the nuclear receptors in the downregulation of the target gene expression. Furthermore, it is also referred to as a silencing mediator for retinoid or thyroid-hormone receptors (SMRT) or T3 receptor-associating cofactor 1 (TRAC-1) [27]. As well, NCOR2 interacts with TBL1X referred to as a subunit in the corepressor SMRT complex along with the histone deacetylase-3 protein [28]. Therefore, these data suggest that it is the binding of DIO1 to NCOR2 and TBL1X to raise its downexpression. However, it should also be noted that DIO2 is an enzyme highly expressed in the thyroid and may significantly contribute to the relative increase in thyroidal production in patients with Graves' disease and thyroid adenomas [29]. DIO2, together with DIO1, catalyzes the removal of an iodine residue from the prohormone thyroxine (T4), producing either the active form triiodothyronine (T3; activation) or the inactive metabolites (reverse T3; inactivation). To this goal, it is also reported that DIO2, as well as DIO1, is downexpressed in nearly all papillary thyroid carcinomas [30], and low levels of DIO1 expression were also demonstrated in the liver carcinoma when compared with normal tissue [31]. As regards DIO1 and DIO2, they are implicated in thyroid metabolism (as reported above); therefore, we can suggest that in HepG2 and Huh7 cell lines there is an impairment of this pathway, consistent with a recent paper where it has been reported that the thyroid hormone receptors promote metastasis of the human hepatoma cells and that the disruption of the cellular thyroid hormone signaling triggers chronic liver diseases, including alcoholic or nonalcoholic fatty liver disease and HCC [32].

All these data suggest that the selenotranscriptome is correlated by means of network analysis with different genes, already reported in the literature as involved in the processes leading to cancer.

In conclusion, in this paper we propose (i) a signature of seleno-mRNAs specific for human hepatoma cells showing the genes that change their expression as a consequence of the liver cancer in the absence of any genetic mutations or viral infection and (ii) the HUB nodes, representing the centers of correlation that exercise a direct control over the coordinated genes.

However, we are planning new studies, which will regard the evaluation of the selenotranscriptome in bioptic tissues of HCC patients to confirm the results obtained by RT-qPCR analysis on HCC cell lines and to suggest new markers to improve the diagnosis and prognosis of this cancer.

## Figures and Tables

**Table 1:** Parameters for RT-qPCR analysis.

Gene	Tm [°C]	Ta [°C]	Sequence (5′ → 3′)
DIO1	*59.8 *	*61 *	AGCTTACTCTGGCTTTGCCGA (21)
			TATTACCCGTCTTCTCGCCCA (21)

DIO2	59.8	60	CTTACTCTGGCTTTGCCGAGA (21)
			CAGGATGTTCCGCTTGACTCT (21)

DIO3	59.8	60	GGTAGTTTCCCCCGCTTGTTT (21)
			TTTAGGTGCTGCTTTGAGGCC (21)

GPX1	59.8	60	TTATGACCGACCCCAAGCTCA (21)
			ATGTCAATGGTCTGGAAGCGG (21)

GPX2	57.3	58	GGAGAATGAACCCAAGCGAA (20)
			CAGGTTTGTCACAGCCAGTGAT (22)

GPX3	59.8	60	TCTCATCCCATGTCCACCATG (21)
			TGCATCCATTTGTGCCAGG (19)

GPX4	59.8	60	AGAGATCAAAGAGTTCGCCGC (21)
			TCTTCATCCACTTCCACAGCG (21)

GPX5	57.9	58	TCCTTCCACGACAATGGTTCA (21)
			TGTGACTGTGACCCCATTGCT (21)

GPX6	59.8	61	CAGAAACCCCACCTCACATGA (21)
			TGCCATGACCTGAATGCACT (20)

GPX7	57.9	56	TTGGTCCCATCATTCTTGTGG (21)
			GGCTGGTGATTCACTGGTCAA (21)

SELI	56.7	59	AAAGGCCAGGTTCCCAGAA (19)
			CAATCCTGCTGCAGTCCAAGT (21)

SELK	*57.3 *	*59 *	AATCAATCATCTGCGTGGCC (20)
			TGGTCAGCCTTCCACTTCTTG (21)

SELM	57.9	61	TCACGCAGGACATTCCATTCT (21)
			CCTGCACTAGCGCATTGATCT (21)

SELO	59.8	60	CGGTTGTGTTGCGTGTAGCTT (21)
			TGCACTCGAATGTCGTTCCTC (21)

SELS	59.8	56	CAGCTGCTCGACTGAAAATGC (21)
			GCATGCTGTCCCACATTTCAA (21)

SELT	57.9	58	TCAATCCCACACCATCGATCA (21)
			ACAACGAGCCTGCCAAGAAAG (21)

SELV	57.9	59	GTGGATTCGTCATTTCCCATG (21)
			TTTGAGTCTGACTGCCATCCC (21)

SEP15	59.8	59	ATCGGAGGCATGCAGAGAGTT (21)
			TCTGCAATCAGGATCCAGCTG (21)

SEPHS2	57.3	60	CGGCTCGCTTTTGTTCTGAA (20)
			TCGCGGCTTGTCAATGATC (19)

SELN	59.8	59	AGGCAGATGCTCATTGTTCCC (21)
			CCCCAAATCCAGATGCAGACT (21)

SEPX1	59.8	61	AGCGGCTGTTGCTCCATAACT (21)
			ATTTCAGCATCACCCACCCTC (21)

TrxR1	57.9	60	CACAATTGGAATCCACCCTGT (21)
			GGTTTGCAGTCTTGGCAACA (20)

TrxR2	*57.9 *	62	AGGACATTTGCTGGTCGAAGC (21)
			GGAATCCCCTGGAAAAACGTT (21)

SEPP1	59.8	57	TAGGAGCTGATGCTGCCATTG (21)
			ATGTTCTCCTCTGCCCGAAGT (21)

SEPW1	59.8	60	GTTTATTGTGGCGCTTGAGGC (21)
			CCATCACTTCAAAGAACCCGG (21)
